# Fabrication and Characteristics of Reduced Graphene Oxide Produced with Different Green Reductants

**DOI:** 10.1371/journal.pone.0144842

**Published:** 2015-12-14

**Authors:** Changyan Xu, Xiaomei Shi, An Ji, Lina Shi, Chen Zhou, Yunqi Cui

**Affiliations:** Packaging Engineering Department, Nanjing Forestry University, Nanjing, Jiangsu, China; Brandeis University, UNITED STATES

## Abstract

There has been an upsurge of green reductants for the preparation of graphene materials taking consideration of human health and the environment in recent years. In this paper, reduced graphene oxides (RGOs) were prepared by chemical reduction of graphene oxide (GO) with three green reductants, L-ascorbic acid (L-AA), D-glucose (D-GLC) and tea polyphenol (TP), and comparatively characterized by X-ray photoelectron spectroscopy (XPS), Fourier transform infrared (FTIR) spectra, Raman spectra and electrical conductivity analysis. Results showed that all these three reductants were effective to remove oxygen-containing functional groups in GO and restore the electrical conductivity of the obtained RGO. The RGO sample with L-ascorbic acid as a reductant and reduced with the existence of ammonia had the highest electrical conductivity (9.8 S·cm^-1^) among all the obtained RGO samples. The mechanisms regarding to the reduction of GO and the dispersion of RGO in water were also proposed. It is the good dispersibility of reduced graphene oxide in water that will facilitate its further use in composite materials and conductive ink.

## Introduction

Graphene, a burgeoning material with a honeycomb-like two-dimensional crystal structure formed by carbon atoms in sp^2^ hybrid junction, exhibits incomparable electronic, thermal, and mechanical properties [1–3]. It has been paid much attention since its first appearance in 2004 when Geim et al. obtained one atomic layer thickness graphene from graphite by peeling with adhesive tape [4]. Graphene presents great potential applications in various fields, including electrodes [5], supercapacitors [6], composite materials [7–8] and sensors [9]. However, it is still a bottleneck to produce high-quality graphene on a large scale and at low cost so as to put graphene into practical applications effectively. There are several methods to obtain graphene, such as mechanical exfoliation [4,10], epitaxial growth [11], liquid-phase exfoliation of graphite [12–13], chemical vapor deposition [14–15], as well as chemical reduction of graphene oxide (GO) [16–18]. Among all these processes, chemical reduction of GO has been regarded as the fastest way to produce graphene in large quantities, and it also has an advantage of solution-processable [19]. This technique involves two steps [20]. First, graphite oxide is obtained by oxidation of graphite powder using the Staudenmaier process or Hummer’s method [21–22]. Second, graphite oxide is exfoliated to graphene oxide by ultrasonication and then various reductants were used to obtain reduced graphene oxide (RGO). Hydrazine [23–24], sodium borohydride and its derivatives [20], oxalic acid [25], as well as sodium hydrosulfite [26] have been reported as reductants to remove oxygen-containing functional groups of GO and thus to restore the conjugate structure of graphene. Considering the ease for functionalization and industrial scale production, as well as the suitability for applications in polymer composites and graphene conductive ink, this paper focused on the solution processable graphene prepared by green reductants. In the next stage, polymer composites and graphene conductive ink will be fabricated and characterized with the obtained RGO.

Some researches confirmed that hydrazine and sodium borohydride had the ability to remove oxygen functionalities of GO. Tung et al. [23] prepared chemically converted graphene sheets with the largest area reported to date (up to 20×40 mm) by reducing GO with pure hydrazine as a reductant. Shin et al. [27] compared the reducing effect of hydrazine and sodium borohydride, and found that with regard to the dose of reductants, 10 mM sodium borohydride was more useful than 50 mM hydrazine for preparation of RGO from GO in lowering the sheet resistance of RGO. However, both hydrazine and sodium borohydride are highly toxic to human beings as well as the environment. In addition, the hydrophobic nature of obtained reduced GO sheet would lead to irreversible agglomerate of RGO in solutions under van der Waals interactions [28]. In recent years, much attention has been paid to exploring environmentally friendly reductants for production of graphene from graphite oxide with good dispersibility in solvents. L-ascorbic acid (L-AA), D-glucose (D-GLC) and tea polyphenol (TP) are water-soluble and mild reductants. The first innocuous and safe reductant, which was comparable to hydrazine as to the deoxygenation of GO, was ascorbic acid (vitamin C), reported by Fernández-Merino et al. [29]. Also, Zhu et al. [30] successfully synthesized chemically converted graphene nanosheets with reducing saccharides (glucose, fructose and sucrose) as reductants. Wang et al. [19] proposed a facile method based on TP to reduce the exfoliated GO in a green tea solution. Considering the operability, low cost and harmlessness to environment, it is very urgent to optimize the reduction process parameters of GO reduction. He et al. [31] compared the reduction rate, density of small sp^2^ domains, degree of reduction, and stability of the reduced GO suspension of L-ascorbic acid, D-fructose, glucose, sucrose, and Na_2_SO_3_. However, there’s no open reports referring to comparative studies of deoxygenation of GO by L-AA, D-GLC and TP. In addition, no comparative researches in open literatures were about the electronic properties of RGOs prepared by these environmental friendly reductants, which is the most important characteristic index for graphene and is the indicator to evaluate the efficiency of GO reduction [32].

In this paper, RGOs were produced from GO, which was synthesized firstly according to the modified Hummer’s method, and then reduced by three green reductants, L-AA, D-GLC and TP. The role of ammonia in L-AA and D-GLC reduction processes was also observed. Reduction processes and electrical properties of RGOs were comparatively investigated by XPS, FTIR spectra, Raman spectra and electrical conductivity analysis. Besides, considering that little literature was about the reduction mechanism of GO by environmentally friendly reductants, we also discussed possible reduction mechanisms of GO by L-AA, D-GLC and TP and their dispersion mechanisms in water with the intention of opening a new perspective in the research of graphene-based composite materials and graphene conductive ink.

## Materials and Methods

Natural graphite powder (40 μm) was obtained from Qingdao Henglide Graphite Co., Ltd. Concentrated sulfuric acid (H_2_SO_4_, 98%), Potassium permanganate (KMnO_4_), Hydrogen peroxide (H_2_O_2_, 30%), Hydrochloric acid (HCl), and ammonia solution were purchased from Nanjing Chemical Reagent Co., Ltd. L-ascorbic acid (L-AA), D-glucose (D-GLC), Potassium persulfate (K_2_S_2_O_8_) and Phosphorus pentoxide (P_2_O_5_) were purchased from Sinopharm Chemical Reagent Co., Ltd. Tea polyphenol (TP, purity of 80%) was generously provided by Ningbo Sinorigin Bio-Products Co., Ltd. All chemicals are of analytical grade and used as received without further purification. Deionized water was prepared by a Milli-Q Plus system (Millipore).

### Synthesis of GO

GO was prepared by the modified Hummers’ method [22,33]. Briefly, graphite powder (10 g) was mixed with K_2_S_2_O_8_ (8.4 g), P_2_O_5_ (8.4 g) and H_2_SO_4_ (60 mL) in a beaker. The mixture was heated to 80°C and kept for 5 h under vigorous stirring. After cooling to room temperature by adding distilled water, the mixture was vacuum-filtered to wash by deionized water on a 0.22 μm Nylon membrane until the insoluble substance was neutral. The solid was vacuum-dried overnight. Then the obtained insoluble matter (4 g) was moved into a beaker filled with H_2_SO_4_ (240 mL) in an ice bath. After slowly adding KMnO_4_ (30 g) under continuous stirring while keeping the temperature below 10°C, the mixture was then heated to 35°C with vigorous stirring and kept for 4 h, followed by being diluted with distilled water (0.5 L) and kept at 50°C for 2 h. Successively, deionized water (1 L) was again added into the mixture, followed by a dropwise addition of H_2_O_2_ (20 mL) under vigorous stirring. With the gradual addition of H_2_O_2_, the color of the mixture gradually turned from dark brown into brilliant yellow. After being filtered with HCl (5 wt%) for 3 times while it was hot, the undissolved substance was washed with deionized water until it was neutral, resulting in graphite oxide. In order to remove the residual salts and acids, the resulting graphite oxide was dialyzed with a molecular weight cut-off membrane (MW 3500 Da) with deionized water for at least 2 weeks at room temperature. Exfoliation of graphite oxide to GO sheets was performed by sonicating the graphite oxide dispersion for 2 h (XO-1200, Xianqu Biological Technology Co., Ltd., China) in the condition of 20 kHz and 80 W. After centrifugation (H-1650, Hunan Xiangyi Laboratory Instrument Development Co., Ltd., China) at 4000 rpm for 30 min to remove the unexfoliated GO sheets, homogeneous GO dispersion was obtained.

### Chemical reduction of GO

L-AA (300 mg) and the obtained GO suspension (300 mL, 0.1 mg·mL^-1^) were mixed, and then ammonia solution (20 μL, 25% w/w) was added to adjust the pH of the suspension to 9–10 to give full play of the colloidal stability of GO sheet through electrostatic repulsion in alkaline conditions [34]. After being sonicated for 30 min, the suspension was heated to 95°C under vigorous stirring and kept for 2 h, resulting in RGO. The produced RGO, designated as L-AA RGO-1, was then centrifuged at 3000 rpm for 15 min, followed by the wash process with distilled water until it was neutral.

The procedure for preparing RGO with D-glucose as a reductant, designated as D-GLC RGO-1, was the same as that for L-AA RGO-1.

The reduction processes without the addition of ammonia solution were also carried out. The resulting RGOs were designated as L-AA RGO-2 and D-GLC RGO-2, respectively.

In order to investigate the role of ammonia solution in the reduction process of GO, ammonia solution (25% w/w) was added to GO suspension (300 mL, 0.1 mg·mL^-1^) to adjust the pH to 9–10. Then the suspension was heated to 95°C under vigorous stirring and kept for 2 h, and the resulting RGO was designated as N RGO. After cooling to room temperature, the suspension was washed with distilled water until it was neutral.

However, the process for the sample reduced by TP, designated as TP RGO, was different. It has been reported that polyphenols are highly sensitive to high temperature and alkaline pH, which will deteriorate the activity of polyphenols [35]. Therefore, ammonia was not used in this case and the reaction temperature was lower than that of L-AA and D-GLC reactions in this paper. Referring to Liao’s method [36], TP (90 mg) was added to GO suspension (300 mL, 0.1 mg·mL^-1^), and then being sonicated for 30 min. After that, the mixture was maintained at 80°C for 8 h. After cooling to the ambient temperature, the resulting suspension was washed with deionized water by filtering through a nylon membrane to remove the residual TP. During this washing process, the faded color of the filtrate suggested the removal of the TP.

**Fig 1:**
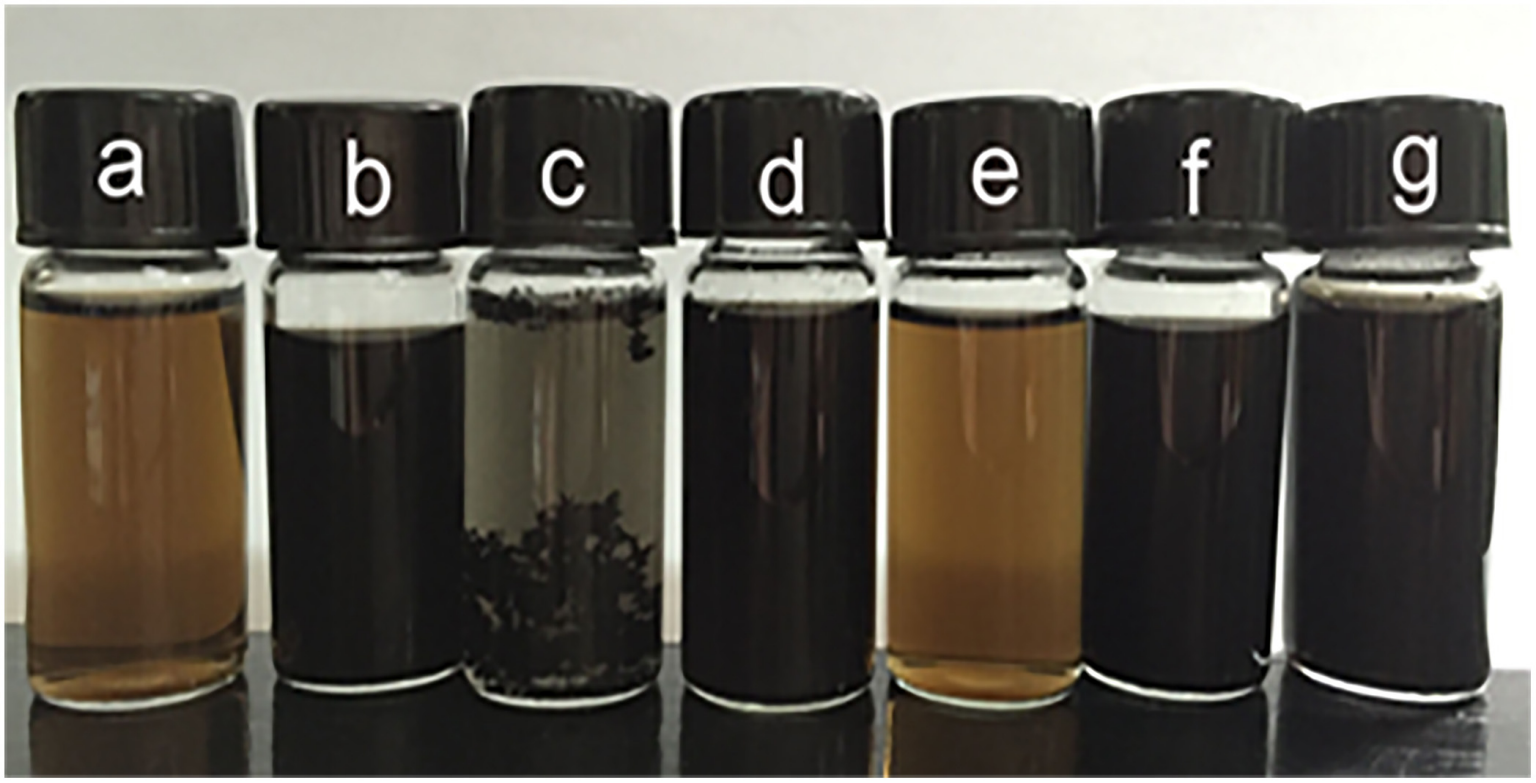
Photograph of the samples (0.1 mg·mL^-1^, GO (a) and RGOs (L-AA RGO-1 (b), L-AA RGO-2 (c), D-GLC RGO-1 (d), D-GLC RGO-2 (e), N RGO (f), and TP RGO (g)).

As shown in Fig 1, it should be noted that except D-GLC RGO-2 (e), the colors of RGOs changed from brown to black, which was the evidence of the reduction process converting GO into RGO [37], and L-AA RGO-2 (c) was agglomerated. The phenomena will be discussed later in this article.

### Characterization

GO, L-AA RGO-1, L-AA RGO-2, D-GLC RGO-1, D-GLC RGO-2, N RGO and TP RGO sample were freeze-dried and then grounded into powder. XPS spectra of graphite, GO and RGO samples were recorded on an AXIS UltraDLD (SHIMADZU, UK) with an Al Kα radiation (1486.6 eV). The FTIR spectra of graphite, GO and RGO samples were observed by a Nicolet iS10 spectrometer (Thermo Scientific Inc., USA) from 4000 cm^-1^ to 500 cm^-1^ at a resolution of 4 cm^-1^. Raman spectra of graphite, GO and RGO samples were observed from 800 cm^-1^ to 2000 cm^-1^ on a Laser Micro-Raman spectroscope (DXR532, Thermo Scientific Inc., USA) using 532 nm He-Ne laser excitation. The electrical conductivities of graphite, GO and RGO samples were tested on a four-point probe system (RTS-8, Guangzhou Four-Point Probes Technology co., Ltd., China). All samples were tableted into “papers” (1 cm in diameter, 0.3 mm in thickness) by a tablet press (YP-2, Shanghai Shang Yue Scientific Instrument co., Ltd., China) under a pressure of 15Mpa, followed by being vacuum-dried at 80°C for 1 h. Electrical conductivity measurements were repeated on at least 3 different areas of the “papers”.

## Results and Discussion

### Chemical reduction degree of GO

**Fig 2:**
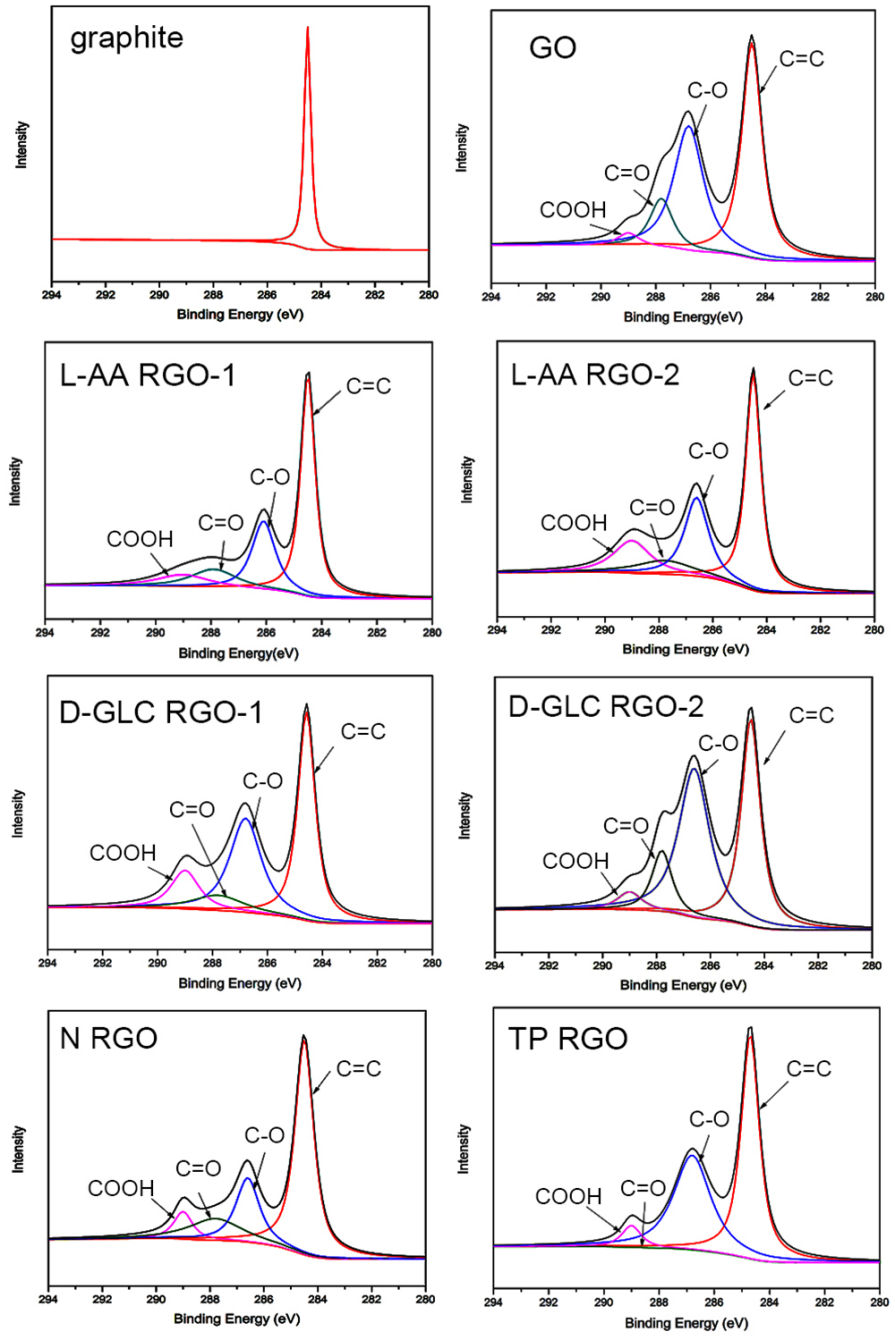
C1s XPS spectra of graphite, GO and RGOs.

XPS is generally used to investigate chemical reduction degree of GO in detail [38]. The C1s XPS spectra of graphite, GO and RGO samples were shown in Fig 2. In Fig 2, the spectrum of graphite presented one sharp peak corresponding to C-C stretching at 284.5 eV, suggesting that no oxygen containing groups exist in pristine graphite. While the spectrum of GO sample presented four deconvolution peaks, corresponding to the C = C/C-C in aromatic rings (284.5 eV), epoxy C (C-O, 286.8 eV), carbonyl C (C = O, 287.8 eV) and carboxyl C (-COOH, 289.0 eV), respectively [32]. For the RGO samples reduced by L-AA with or without the addition of ammonia, the intensity of peaks assigned to oxygen-containing functional groups decreased significantly after reduction, indicating a considerable deoxygenation of GO. However, the intensity of COOH in the C1s spectrum of L-AA RGO-2 was higher than that of GO. When L-AA was used as a reductant without the addition of ammonia, the COOH group in decomposition product of L-AA (oxalic acid) [39] cannot be neutralized by ammonia, which will be responsible for a higher intensity of COOH in L-AA RGO-2. Despite some oxygen group peaks existing in the C1s spectra of D-GLC RGO-1, N RGO and TP RGO, the peak intensity was greatly smaller than that of the GO sample. It should not be neglected that there’s no significant difference between the spectrum of D-GLC RGO-2 and GO, demonstrating that weak reduction was implemented by D-GLC in the absence of ammonia. In addition, it’s obvious to find that the peak intensity of carboxyl C in the spectrum of D-GLC RGO-1 was much higher than that of D-GLC RGO-2. The oxidization of the aldehyde group in glucose by GO in alkaline solution can produce carboxyl group [30], which further verified that the existence of ammonia can allow full play to the reducibility of D-GLC.

In the XPS study of carbon materials, FWHM (Full Wave at Half Maximum) of C = C FWHM_(C = C)_ is usually negatively related to the conductivity of the materials, that is the lowest FWHM_(C = C)_ corresponding to the highest conductivity [40]. Table 1 showed the values of FWHM_(C = C)_ of graphite, GO and RGO samples. The decrease of FWHM_(C = C)_ after reduction indicated the increase in conductivity of the samples by restoration of sp^2^ network. Meanwhile, the obvious intensity decrease of the peak C-O in the spectra of RGO samples in Fig 2 also implied the increase in conductivity, which is in line with Obata’s research [32].

**Table 1:** FWHM_(C = C)_ value and C/O atomic ratio of graphite, GO and RGOs.

Samples	graphite	GO	L-AA RGO-1	L-AA RGO-2	D-GLC RGO-1	D-GLC RGO-2	N RGO	TP RGO
**the FWHM**_**(C = C)**_ **value**	0.34	0.88	0.63	0.68	0.65	0.85	0.74	0.77
**C/O atomic ratio**	17.87	2.65	5.15	4.87	2.89	2.67	3.60	3.44

In XPS investigation, the change of C/O atomic ratio of RGO suggests the reduction degree [38]. As shown in Table 1, the ratio derived from the XPS data showed various degree of increases after reduction, demonstrating that most oxygen-functionalized groups were removed during reduction process. As for graphite, its C/O atomic ratio was 17.87, which was in agreement with the report by Moon et al. [41]. The C/O atomic ratios of L-AA RGO-2 and D-GLC RGO-2 were a little lower than that of their corresponding RGO-1. In the absence of ammonia, deoxygenation of GO by L-AA and D-GLC was less effective, which was consistent with the observation that intensities of C-O and C = O peaks in RGO-2 were higher than those in RGO-1. And RGOs reduced by L-AA with or without the addition of ammonia showed much higher C/O atomic ratio than the other RGOs, which indicating that a better deoxygenation of GO can be achieved by L-AA. However, C/O atomic ratio of L-AA RGO-1 (5.15) was lower than that of RGO reduced by vitamin C (~12.5) [29]. It can be deduced that the difference of C/O atomic ratio may be ascribed to not only a few differences between the production processes of GO in two researches, but also the different content of GO used in the reduction process. Some oxygen groups remaining on the RGO surface may be benefit for chemical functionalization in preparation of composite materials and conductive ink in the next stage. For all RGO samples in this research, C/O atomic ratios were lower than that of RGO reduced by hydrohalic acid (about 12) [16]. However, hydrohalic acid is corrosive and harmful to human being and the environment. Some residual oxygen groups still exist in RGOs, showing the intrinsic defect of RGO. This result was consistent with the former report [29].

### Changes of functional groups in the reduction process

**Fig 3:**
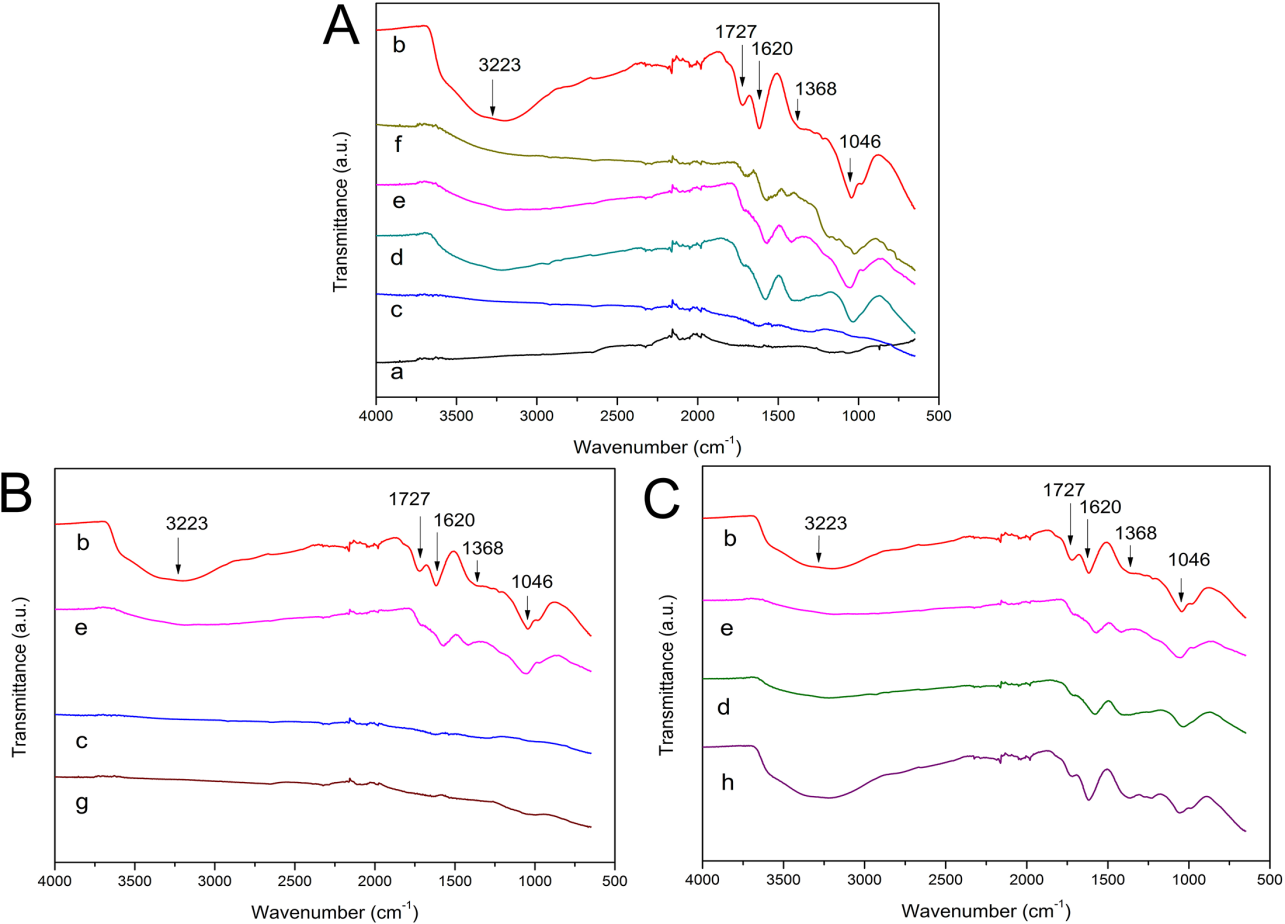
FTIR spectra of graphite (a), GO (b) and RGOs (L-AA RGO-1 (c), D-GLC RGO-1 (d), N RGO (e), TP RGO (f), L-AA RGO-2 (g) and D-GLC RGO-2 (h)).

FTIR spectroscopy is regarded as an important tool for characterization of functional groups [42]. The FTIR spectra of graphite and its corresponding GO, RGOs with different preparation methods were shown in Fig 3A, and the specific comparative FTIR spectra of RGOs prepared with or without ammonia and N RGO were shown in Fig 3B and 3C. In Fig 3A, the characteristic peaks at 3223, 1727, 1620, 1368, 1221, and 1046 cm^-1^ in the GO spectrum (b) were due to O-H stretching, C = O stretching, aromatic C = C stretching, O-H deformation, epoxy C-O stretching, and alkoxy C-O stretching vibrations [30,43], respectively. These peaks did not appear in the spectrum of graphite sample (a), indicating that the oxidation step introduced a large number of oxygen-containing functional groups, and these groups should include -COOH and C = O located at the sheet edge, -OH and epoxy C-O on the basal planes of the GO sheet [44]. After reduction by L-AA with the addition of ammonia (c), almost all the characteristic peaks weakened and some even disappeared, which was consistent with the result of the XPS analysis in this paper. The curve of L-AA RGO-1 was similar to that of graphite, showing the restoration of electronic conjugation within graphene sheets. In the spectra of D-GLC RGO-1 (d) and TP RGO (f), the intensity of peaks at 3223, 1727, and 1368 cm^-1^ decreased dramatically, demonstrating the removal of oxygen-containing functional groups to a certain degree. In the spectrum of N RGO (e), the intensity of oxygen-containing functional groups decreased to some extent, and this result was consistent with Fan’s report [34]. Besides, the existing C = C peak at 1620 cm^-1^ in the spectra of all RGO samples suggested that the sp^2^ structure of carbon atoms was remained, and this result was in line with the finding of SU et al. [45]. In Fig 3B, no significant differences presented between the spectra of L-AA RGO-1 (c) and L-AA RGO-2 (g). It demonstrated that the addition of ammonia in suspension had little influence on the functional groups of resulting RGO; however, the existence of ammonia would prevent the agglomeration of graphene sheets to a great extent, which was proved by the well-dispersed suspension of L-AA RGO-1 in Fig 1. In Fig 3C, the intensity of oxygen-containing functional groups in D-GLC RGO-1 (d) decreased more considerably than that in D-GLC RGO-2 (h), indicating the weak reducibility of D-GLC in the absence of ammonia. Considering this result and the different colors of D-GLC RGO-1 and D-GLC RGO-2 shown in Fig 1, it can be deduced that the synergistic effect caused by the introduction of ammonia benefited the deoxygenation of GO, and this result was in line with the result of the XPS analysis in this paper and previous researches [30,34]. So, a conclusion can also be drawn that the reducing ability of L-AA was the highest among all the used reductants according to the removal of oxygen-containing functional groups, and the addition of ammonia would prevent resultant RGO from agglomerating.

### Raman spectroscopy investigation of the samples

**Fig 4:**
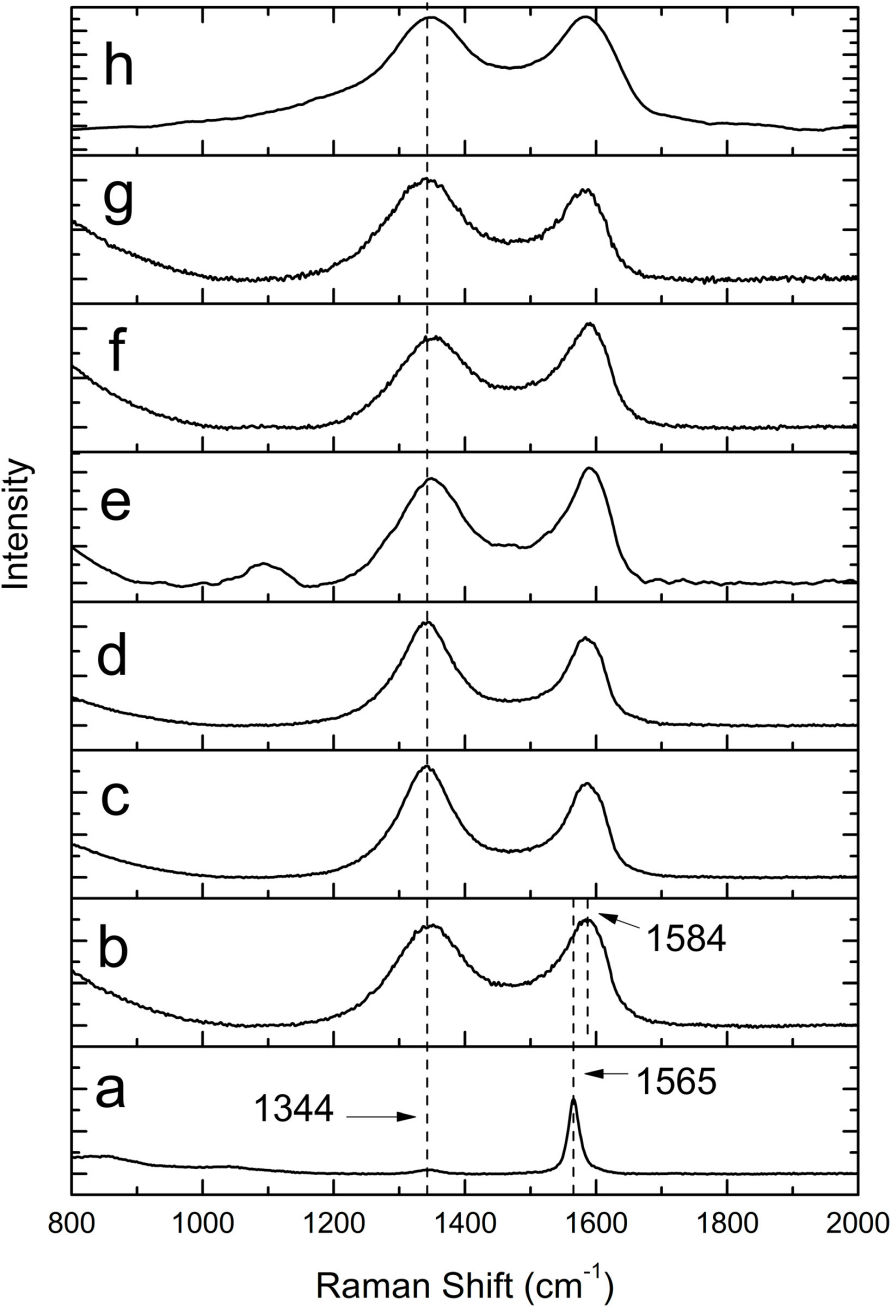
Raman spectroscopy spectra of graphite (a), GO (b) and RGOs (L-AA RGO-1 (c), L-AA RGO-2 (d), D-GLC RGO-1 (e), D-GLC RGO-2 (f), N RGO (g), and TP RGO (h)).

Raman spectroscopy is one of the most common and effective techniques for analyzing the structure changes of graphene-based materials, including disorder and defect structures, defect density, and doping levels [30,46]. Two prominent features are usually observed in the Raman spectra of graphene, namely the G band (~1580 cm^-1^) and the D band (1270–1450 cm^-1^, depending on laser wavelength [47]). The G band, relating to the graphite carbon structure, is corresponding to the first order scattering of E_2g_ phonon of sp^2^ C atoms at the Brillouin zone center, while the D band, indicating typical defects attributed to the structural edge effects, is arising from a breathing mode of rings or K-point photons of A_1g_ symmetry [3,30,48]. Fig 4 showed the Raman spectroscopy spectra of graphite, GO and RGOs prepared with different reductants. Compared with the spectrum of graphite, a broad G band and a blue-shift (from 1565 cm^-1^ in the spectrum of graphite to 1584 cm^-1^ in that of GO) were observed in the spectrum of the GO sample because of the isolated double bonds resonate at higher frequencies than the G band of graphite [49], indicating that the oxidation took place. Meanwhile, the intensity of D band (1344 cm^-1^) increased dramatically, which was ascribed to the extensive oxidation introducing defects in graphite [50]. After reduction, the spectra of RGO samples with different reductants had similar peaks, however they showed different intensity ratio of the D to G bands (I_D_/I_G_), which is usually an important parameter to characterize the disorder or the extent of covalent modification of the graphene surface [51]. Different I_D_/I_G_ values of GO and RGO samples were shown in Table 2. Compared to GO, all RGO samples except D-GLC RGO-2 presented increased I_D_/I_G_ values. It indicated that the reduction step removed most of the oxygenated functional groups and a reduction in the average size of sp^2^ domains [43,52]. L-AA RGO-1 sample had the highest I_D_/I_G_ value (1.19), showing that the reduction process using L-AA RGO as a reductant with the addition of ammonia produced the most abundant and smallest graphitic domains among all samples. This finding was in accordance with the result of He et al. [31]. The lower I_D_/I_G_ value of D-GLC RGO-2 indicated that insufficient reduction was carried out without the addition of ammonia, which was consistent with the XPS and FTIR analysis in this paper and the report of Venkanna [53]. Loryuenyong et al. [54] reported that they prepared RGO with I_D_/I_G_ value of 1.12 only by stirring and heating GO in distilled water at 95°C for 4 days. Herein when L-AA was used as a reductant, it only took 2 hours to reduce GO and the I_D_/I_G_ value of the resulting L-AA RGO-2 was up to 1.17, showing that L-AA as a reductant to prepare RGO can largely save time and energy. As regards D-GLC, a similar result was obtained. The I_D_/I_G_ value of the RGO produced only using D-GLC (D-GLC RGO-2) was 0.86; however, with the help of synergistic effects caused by the introduction of ammonia, the I_D_/I_G_ value of the RGO (D-GLC RGO-1) was increased to 1.1, which was close to that of the RGO fabricated by Loryuenyong et al. The increasing values of I_D_/I_G_ for all RGO samples compared with GO’s in our experiment were lower than those of He’s finding, which demonstrated that the RGO samples produced in this experiment were less-defected, corresponding to the disorder characteristic for the D band. Also, L-AA RGO samples had better restoration of sp^2^ network than RGOs reduced by D-GLC and TP in reference to XPS analysis. Therefore, L-AA is the most effective reductant for GO to obtain RGO among the used reductants in this study.

**Table 2:** Different values of I_D_/I_G_ of the samples.

Samples	GO	L-AA RGO-1	L-AA RGO-2	D-GLC RGO-1	D-GLC RGO-2	N RGO	TP RGO
**I**_**D**_ **/I**_**G**_	0.95	1.19	1.17	1.1	0.86	1.09	0.98

### Electrical conductivity of the obtained reduced graphene oxide

Previous research has revealed that GO shows electrical insulation and chemical reduction can remove oxygen-containing functional groups to regain electrical conductivity of RGO [49]. Electrical conductivity of reduced graphene oxide has usually been used to indicate the restoration extent of electronic conjugation in deoxidating GO [29]. As shown in Table 3, the electrical conductivity of GO was quite low, and 5 orders of magnitude lower than that of graphite, indicating its electrical insulation. Meanwhile, there was a great disparity among the conductivity values of RGO samples prepared by different reductants, revealing that the conductivity of the obtained reduced graphene oxide was greatly influenced by reduction process and reductants. This result was in good agreement with the statement of Zhao et al. [55]. In our experiment, the L-AA RGO-1 showed the best conductivity among all the obtained RGO samples, 9.8 S·cm^-1^, which was much higher than that of RGO sample produced by Gao et al. (0.141 S·cm^-1^) [37]. The addition of ammonia provided the possibility of GO deoxygenating to some extend [34], which will be beneficial to the increment of electronic conductivity of resulting RGO. In addition, L-tryptophan, which Gao et al. used as a stabilizer to avoid the agglomeration and precipitation of the resulting graphene sheets, would be absorbed on the surface of reduced graphene oxide, resulting in the degradation of electronic property of the obtained RGO. As for L-AA RGO-2 prepared without the addition of ammonia, the electrical conductivity was a bit lower than that of L-AA RGO-1. Under the condition of no electrostatic stabilization provided by alkali media, RGO would aggregate and the aggregation would further hinder the complete interconnection between conductive paths, resulting in weaker electronic conductivity. In Table 3, the D-GLC RGO-2 sample presented the lowest conductivity value compared with other samples due to the weak reducibility of D-GLC without the synergistic effect offered by ammonia. This phenomenon was discussed in the XPS, FTIR and Raman analysis in this research. Meanwhile, the impressive electronic conductivity of N RGO (3.73) also proved Fan’s opinion that simply heating GO suspension under strongly alkaline conditions can be a green route to prepare graphene [34]. The conductivity value of TP RGO was 1.36 S·cm^-1^, pretty lower than that of RGOs prepared by L-AA, D-GLC (with the addition of ammonia) and ammonia. Wang et al. [19] believed that the adsorbed TP molecules on the graphene surface limited the conductivity of TP RGO. In addition, the conductivity value, 1.36 S·cm^-1^, was comparatively lower than that of Liao (23.85 S·cm^-1^) [36], which may be caused by the fact that the purity of tea polyphenol they used was higher than ours. Furthermore, I_D_/I_G_ value of TP RGO in this paper was lower than that of Liao, indicating less oxygenated functional groups were removed in our case, corresponding to lower electrical conductivity. When it comes to the restoration of the π-conjugated structure, D-GLC (with the addition of ammonia) was somewhat more efficient than TP, but not as good as L-AA. This result was in line with the findings of XPS, FTIR analysis as well as Raman analysis in our experiment.

**Table 3:** Conductivity of the obtained RGO samples.

Samples	graphite	GO	L-AA RGO-1	L-AA RGO-2	D-GLC RGO-1	D-GLC RGO-2	N RGO	TP RGO
**Conductivity (S·cm** ^**-1**^ **)**	28±10	1.56×10^−4^ ±1.75×10^−5^	9.8 ±0.63	7.29 ±0.70	3.15 ±0.34	3.53×10^−4^ ±8.43×10^−5^	3.73±0.19	1.36±0.22

### Possible reduction mechanism of GO and dispersion mechanism of RGO

**Fig 5:**
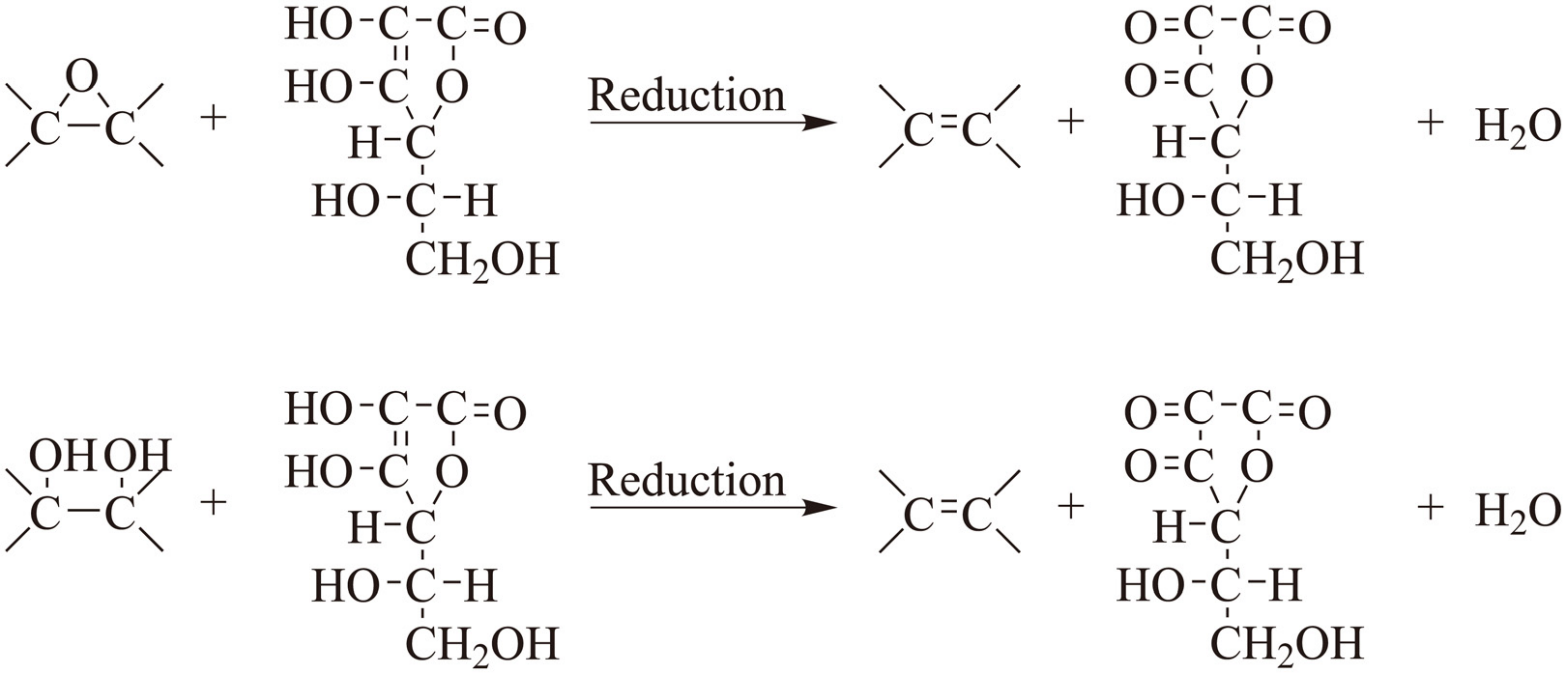
Possible reaction mechanism of GO reduction by L-AA.

Fig 5 represented the possible reaction mechanism of GO reduced by L-AA. On the basis of aforementioned XPS and FTIR analysis, hydroxyl and epoxide groups on the basal planes of the GO sheets eliminated, indicating that those groups reacted with hydrogen atoms in the 5-membered ring of L-AA to yield H_2_O. During the reduction process, SN2 nucleophilic attack and subsequent thermal elimination will restore the C = C bond, which indicates that graphene oxide has been converted to reduced graphene. And acidic L-AA is easy to deprotonate to form dehydroascorbic acid [56], which will further be converted into guluronic acid and oxalic acid [39]. And the intermediate acids can be neutralized by ammonia to prevent the accumulation of dehydroascorbic acid products so as to facilitate the reduction process [31]. Meanwhile, the alkaline condition may offer electrostatic repulsion between RGO sheets [34] and the intermediates may form hydrogen bonds with the residual oxygen groups, including -COOH on the edge of the GO sheets, which will hinder the π-π stacking between the RGO sheets and prohibit the formation of aggregations, resulting in a stable L-AA RGO suspension [39].

**Fig 6:**
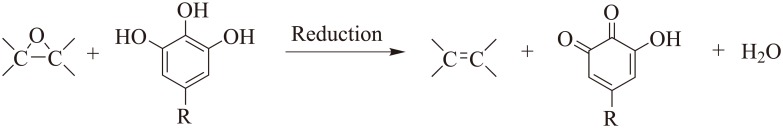
Possible reaction mechanism of GO reduction by TP (R in the chemical equation represent the other structures in EGCG for simplification in illustration purpose).

The reduction mechanism of TP is similar to that of L-AA. Epigallocatechin gallate (EGCG), accounting for 50–60 wt% of TP [19], is the most abundant component in TP and gives TP the unique reducibility. As shown in Fig 6, through an SN2 nucleophilic attack, the oxygen anion of EGCG will open the ring of epoxide on GO sheets, which was proved by the phenomenon that the intensity of epoxy C was decreased in XPS analysis. And a backside SN2 nucleophilic attack will form intermediate and yield H_2_O, resulting in reduced graphene and galloyl-derived orthoquinone after thermal elimination. The oxidized TP strongly absorbed on RGO leading to π-π interactions, which is responsible for the stability of TP RGO suspension [36].

**Fig 7:**
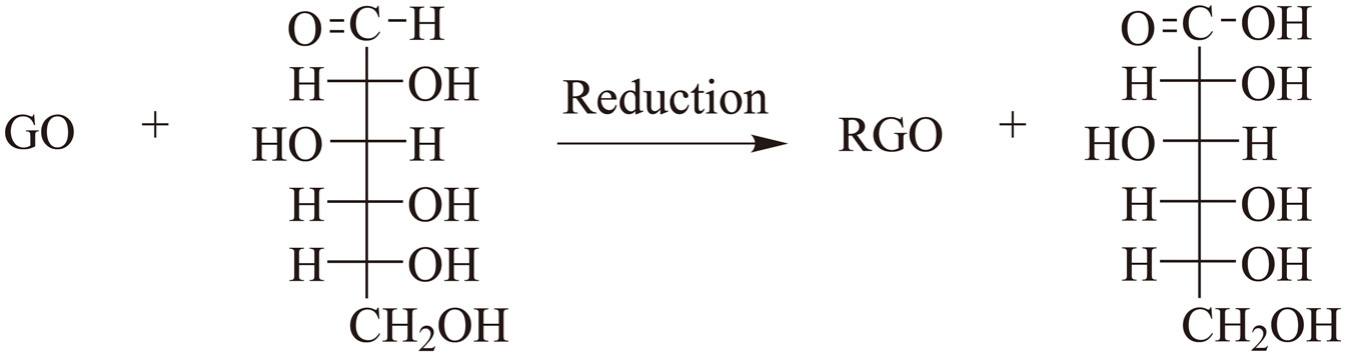
Possible reaction mechanism of GO reduction by D-GLC.

Different from L-AA and EGCG, D-GLC cannot deprotonate to form nucleophile. With the existence of both aldehyde group and hydroxyl group in its molecule, D-GLC can form a ring hemiacetal in the interior of the molecule. Therefore, the ring-like structure and the open chain structure coexistence in D-GLC solution. As shown in Fig 7, the reducibility of D-GLC lies in aldehyde group on the open chain structure, which can be oxidized to carboxyl group by GO in the presence of ammonia. In this case, GO can be seen as the oxidant, and ammonia played the role of stimulator to oxidization process. This process turns glucose into aldonic acid, which can be converted into lactone containing a large number of hydroxyl groups and carboxyl groups, corresponding to the increasing peak intensity of carboxyl C in D-GLC RGO-1 in XPS analysis. The oxidized products of D-GLC can form hydrogen bonds with residual oxygen groups on the GO sheets, preventing the formation of agglomerates, thus resulting in stable D-GLC RGO suspension [30].

## Conclusions

There has been an upsurge of environmentally friendly reductants for preparation of graphene materials. In this research, the reducibility of three green reductants, L-ascorbic acid, D-glucose and tea polyphenol, for graphene oxide was comparatively investigated. The process involved preparation of graphene oxide and its chemical reduction with different reductants to obtain reduced graphene oxide. XPS, FTIR, Raman and electrical conductivity analysis showed that all reductants except D-GLC without the addition of ammonia were effective to convert GO into RGO. The RGO sample using L-ascorbic acid as a reductant with the existence of ammonia had the highest electrical conductivity, 9.8 S·cm^-1^, among all the obtained RGO samples. The existence of ammonia in the reduction process of L-AA provides the possibility of deoxygenation in GO to some extend and prevents the agglomeration of resulting RGO sheets. However, in the reduction process of D-GLC, ammonia offers the synergistic effect to stimulate to the oxidization process, so as to give full play to the activity of aldehyde group on the open chain structure in D-GLC. Possible reduction mechanisms of GO by L-AA and TP are due to SN2 nucleophilic reaction, while the aldehyde group on the open chain structure of D-GLC gives D-GLC the unique reducibility. The good dispersibility of resulting RGOs in water will facilitate the further use of graphene in a larger range of possible applications.
